# How do SGLT2 inhibitors protect the kidney? A mediation analysis of the EMPA-REG OUTCOME trial

**DOI:** 10.1093/ndt/gfae032

**Published:** 2024-02-06

**Authors:** Christoph Wanner, Masaomi Nangaku, Bettina J Kraus, Bernard Zinman, Michaela Mattheus, Stefan Hantel, Martin Schumacher, Kristin Ohneberg, Claudia Schmoor, Silvio E Inzucchi

**Affiliations:** Department of Medicine, Würzburg University Clinic, Würzburg, Germany; Division of Nephrology and Endocrinology, Department of Hemodialysis and Apheresis, The University of Tokyo Hospital, Tokyo, Japan; Medical Affairs, Boehringer Ingelheim International GmbH, Ingelheim, Germany; Department of Internal Medicine I, University Hospital Würzburg, Würzburg, Germany; Comprehensive Heart Failure Centre, University of Würzburg, Würzburg, Germany; Lunenfeld-Tanenbaum Research Institute, Mount Sinai Hospital, Toronto, ON, Canada; Biostatistics, Boehringer Ingelheim Pharma GmbH & Co. KG, Ingelheim, Germany; Biostatistics, Boehringer Ingelheim Pharma GmbH & Co. KG, Biberach, Germany; Institute for Medical Biometry and Statistics and Clinical Trials Unit, Faculty of Medicine, and Medical Center, University of Freiburg, Freiburg, Germany; Institute for Medical Biometry and Statistics and Clinical Trials Unit, Faculty of Medicine, and Medical Center, University of Freiburg, Freiburg, Germany; Clinical Trials Unit, Faculty of Medicine and Medical Center, University of Freiburg, Freiburg, Germany; Section of Endocrinology, Yale University School of Medicine, New Haven, CT, USA

**Keywords:** empagliflozin, kidney disease, mediation analysis, sodium glucose co-transporter, type 2 diabetes

## Abstract

**Introduction:**

Mechanisms underlying kidney benefits with sodium-glucose cotransporter-2 (SGLT2) inhibition in heart failure and/or type 2 diabetes (T2D) with established cardiovascular disease are currently unclear.

**Methods:**

We evaluated *post hoc* the factors mediating the effect of empagliflozin on a composite kidney outcome (first sustained estimated glomerular filtration rate ≥40% reduction from baseline, initiation of renal replacement therapy or death due to kidney disease) in EMPA-REG OUTCOME (Empagliflozin Cardiovascular Outcome Event Trial in Type 2 Diabetes Mellitus Patients). Variables, calculated as change from baseline or updated mean, were evaluated as time-dependent covariates and using a landmark approach (at Week 12) in Cox regression analyses. In multivariable analyses, variables with the greatest mediating effect were added using a step-up procedure.

**Results:**

In univariable time-dependent updated mean covariate analyses, the strongest mediator was hematocrit (99.5% mediation). Hemoglobin, uric acid and urine albumin-to-creatinine ratio mediated 79.4%, 33.2% and 31.0%, respectively. Multivariable analyses were not performed due to the very strong mediation effect of hematocrit. In univariable Week 12 landmark change from baseline analyses, the strongest mediators included hematocrit (40.7%), glycated hemoglobin (28.3%), systolic blood pressure (16.8%) and free fatty acids (16.5%), which yielded a combined mediation of 78.9% in multivariable analysis.

**Conclusions:**

Changes in hematocrit and hemoglobin were the strongest mediators of empagliflozin's kidney benefits in EMPA-REG OUTCOME participants with T2D and cardiovascular disease.

KEY LEARNING POINTS **What was known:**

- Sodium-glucose cotransporter-2 (SGLT2) inhibitors have demonstrated kidney benefits in clinical trials across a broad range of patients with cardio-renal-metabolic diseases.
- In the EMPA-REG OUTCOME trial, the SGLT2 inhibitor empagliflozin reduced the risk of incident or worsening nephropathy by 39%, and the risk of a composite kidney outcome by 46%, compared with placebo in patients with type 2 diabetes and established cardiovascular disease.
- Despite extensive research, the mechanisms underlying the kidney benefits seen with SGLT2 inhibitors remain undetermined.

**This study adds:**

- In this *post hoc* mediation analysis of the EMPA-REG OUTCOME trial, changes in hematocrit and hemoglobin levels were the strongest mediators of the empagliflozin treatment kidney benefit.

**Potential impact:**

- These findings complement previous analyses on the reduction in cardiovascular mortality and hospitalizations for heart failure seen with empagliflozin, showing that markers of plasma volume and/or red blood cell mass appear to mediate to a large degree the cardio-kidney organ protection in the EMPA-REG OUTCOME trial.
- The predominance of hematocrit and hemoglobin as mediators of empagliflozin's kidney benefits may suggest an erythropoietic mechanism of action, potentially linked to the alleviation of organ hypoxia.
- Further studies are required to validate the underlying mechanisms suggested by the findings of this study.

## INTRODUCTION

In the EMPA-REG OUTCOME (Empagliflozin Cardiovascular Outcome Event Trial in Type 2 Diabetes Mellitus Patients) trial, the sodium-glucose cotransporter-2 (SGLT2) inhibitor empagliflozin reduced the risk of incident or worsening nephropathy by 39%, and the composite kidney outcome of doubling of serum creatinine, initiation of renal replacement therapy or death due to kidney disease by 46% versus placebo in patients with type 2 diabetes (T2D) and established cardiovascular disease [1]. Studies have also suggested that empagliflozin reduces the risk of adverse kidney outcomes across a wide range of kidney disease stages in patients with T2D and cardiovascular disease [2], and also in those with heart failure and/or chronic kidney disease (CKD) irrespective of the presence of T2D [3]. More recently, the CREDENCE, DAPA-CKD and EMPA-KIDNEY studies have demonstrated that SGLT2 inhibitors reduce kidney disease progression in patients with CKD [4–6]. DAPA-CKD and EMPA-KIDNEY included a substantial proportion of patients without T2D and reported consistent benefits regardless of presence of diabetes. This finding is supported by a meta-analysis of SGLT2 inhibitor studies [7]. However, the underlying mechanisms of these highly impactful effects remain incompletely understood.

A number of potential mechanisms underlying the kidney benefits of SGLT2 inhibitors have been proposed, including: activation of tubuloglomerular feedback (leading to reduction of intraglomerular pressure and glomerular hyperfiltration) [8]; reduced proximal tubule metabolic stress and hypoxia [9]; reduced mitochondrial damage [10]; reduced hyperglycemia-induced inflammation, reduced oxidative phosphorylation and less production of reactive oxygen species and angiotensinogen [11–13]; and improved oxygenation/reduction in plasma volume [14, 15]. In this study, we investigated the mechanisms underlying the kidney benefits observed with empagliflozin by *post hoc* mediation analysis of EMPA-REG OUTCOME trial data.

Mediation analyses can be used to investigate the mechanisms underlying the effect of one variable on another, such as the treatment effect of a drug on a clinical outcome, by statistically assessing the degree to which a variable or combination of variables contribute(s) to the observed effect [16]. In a *post hoc* mediation analysis of EMPA-REG OUTCOME trial data, markers of plasma volume and/or red blood cell mass were shown to be the strongest mediators of the reduction in cardiovascular mortality observed with empagliflozin (hematocrit and hemoglobin mediated 51.8% and 48.9%, respectively) [17]. Similarly, hematocrit and hemoglobin were found to be the most important mediators of the reduction in hospitalization for heart failure and death from heart failure with empagliflozin, with 50.7% and 53.5% mediation, respectively [18].

In this study, we investigated *post hoc* the mechanisms underlying the kidney benefits observed with empagliflozin by mediation analysis of the EMPA-REG OUTCOME trial data.

## MATERIALS AND METHODS

The study design of the EMPA-REG OUTCOME trial has been published previously [19], but in brief, 7020 patients with T2D and glycated hemoglobin (HbA1c) 7.0%–9.0% for drug-naïve patients and 7.0%–10.0% for those on stable glucose-lowering therapy, estimated glomerular filtration rate (eGFR) ≥30 mL/min/1.73 m^2^ and established atherosclerotic cardiovascular disease (defined as prior myocardial infarction, stroke, coronary artery disease or peripheral artery disease) were randomized (1:1:1) to receive empagliflozin 10 mg, 25 mg or placebo, in addition to standard of care (ClinicalTrials.gov: NCT01131676).

*Post hoc*, we investigated potential mediators of the treatment effect of pooled empagliflozin doses (10 mg or 25 mg, once daily) versus placebo on the composite kidney outcome of first sustained eGFR (using the Modification of Diet in Renal Disease Study equation) reduction of ≥40% from baseline, initiation of renal replacement therapy or death due to kidney disease in participants for whom data on all potential mediators were available. First sustained eGFR reduction of ≥40% was confirmed with two consecutive measurements, at least 4 weeks apart. Renal events were captured either on the basis of standardized laboratory assessment or investigator reporting. Renal endpoints were not adjudicated. Initiation of renal-replacement therapy was based on investigator reported dialysis or renal transplant based on 15 preferred terms. For definition of death from kidney disease the following conditions had to be fulfilled: firstly, the patient had an eGFR <10% at any time or the patient experienced one event of broad MedDRA Standardised Medical Query (SMQ) acute renal failure (including nephrotic syndrome), that was considered as leading to death by the investigator; secondly, one of either of the following: the patient was not on dialysis at any time from randomization, or the patient was on dialysis, but stopped with last dialysis occurring at least 14 days before the date of death; thirdly, the patient did not die from cardiovascular death.

We used a traditional mediation analysis as originally proposed by Baron and Kenny [16], taking into account the time-dynamic development of the variable and the composite kidney outcome. In a traditional mediation analysis, a variable must satisfy three conditions to be considered a mediator of the treatment effect. Firstly, a variable can only be regarded a mediator if treatment has an effect on the variable over time. Secondly, the change in the variable over time is required to have an effect on the outcome being assessed. Lastly, the treatment effect [represented as the hazard ratio (HR)] must be reduced (i.e. closer to 1.0) in an analysis that includes adjustment for the variable compared with the treatment effect in an unadjusted analysis.

In a study investigating mediators of the empagliflozin treatment effect on cardiovascular death in the EMPA-REG OUTCOME trial, Inzucchi *et al.* presented an analysis of the treatment effect on several variables over time to assess the first condition for a variable to be considered a potential mediator [17]. Based on the different potential underlying mechanistic processes of the empagliflozin kidney benefits, we selected 17 of the factors identified by the investigators: glycemia [HbA1c and fasting plasma glucose (FPG)]; vascular tone [systolic blood pressure (SBP), diastolic blood pressure (DBP) and heart rate]; lipids [high-density lipoprotein cholesterol (HDL) and low-density lipoprotein cholesterol (LDL), triglycerides and free fatty acids (FFA)]; kidney [urine albumin-to-creatinine ratio (UACR)]; adiposity [weight, body mass index (BMI) and waist circumference]; volume status and hematopoiesis/oxygenation (hematocrit, hemoglobin and albumin); and other (uric acid).

We analyzed the association of each of these variables as a time-dependent covariate with the composite kidney outcome (i.e. the second mediation condition) in Cox regression models using two approaches for the time-dependent covariate: (i) current change from baseline to the most recent value available before the first event of the composite kidney outcome (“current change”); (ii) mean value considering all prior values (“updated mean”). Analysis of the current change represents the current effect of the variable on the risk of the outcome. Analysis of the updated mean represents the cumulative effect of all prior values of the variable on the risk of the outcome. The models included treatment group, baseline value of the variable and the current change or updated mean of the variable as time-dependent covariates. The empagliflozin treatment groups (10 mg and 25 mg) were pooled in all analyses. We present estimated HRs for the composite kidney outcome associated with a 1-unit increase of each variable. Variables were considered to have an effect on the outcome (i.e. the second mediation condition) if the 95% confidence intervals (CI) of the HR did not include 1. Only variables that were found to be significantly associated with the composite kidney outcome were included in subsequent analyses.

We subsequently assessed whether variables fulfilled the third mediation condition by analyzing whether the treatment effect of empagliflozin on the composite kidney outcome was reduced, i.e. HR for treatment was closer to 1.0, in a model adjusted for the variables of change from baseline or updated mean and baseline value, compared with a model adjusted for treatment group alone. Percentage mediation was calculated as described in Inzucchi *et al.* [17].

Further analyses were conducted to assess the joint contribution of various variables to the empagliflozin treatment effect on the composite kidney outcome in a multivariable approach. As described previously [17], this step-up procedure for multivariable model building was based on a ranking of the various variables and their assigned mechanistic categories with regard to their potential as mediators. Variables with the greatest mediating effect based on the univariable analyses were sequentially added using a step-up procedure. From each mechanistic category only the variable with the largest mediating effect was chosen because variables pertaining to the same physiologic category may be biologically and statistically redundant.

As an alternate methodological approach, we performed “landmark” Cox regression analyses, wherein each of the variables was analyzed as a time-fixed covariate at Week 12 (using current change from baseline or updated mean), along with the variable baseline value as a covariate and a factor for treatment group. Following the assessment of the mediation conditions, the multivariable analyses were repeated for this approach. This approach was based on the idea that the major influence of empagliflozin on the potential mediators would have occurred within the first 12 weeks of treatment. Analyses included all patients with available Week 12 values of the variables who were at risk of experiencing the event, which consisted of 6832/6968 patients (98% of the study population). These landmark analyses considered the effect of the early (within 12 weeks) change of the potential mediators on the composite kidney outcome.

The time-dependent analyses investigating the updated mean of the variables were performed as the main approach for considering the cumulative effect of the long-term development of the potential mediators on the composite kidney outcome, whereas the landmark analyses investigating the change from baseline to Week 12 were performed as the main approach for considering the early effect of the immediate change of the potential mediators on the composite kidney outcome. Both other approaches (time-dependent with current change and landmark Week 12 with updated mean) were performed as sensitivity analyses. The sensitivity analyses were used to further probe the robustness of our findings.

Finally, to analyze the statistical stability of the results, a bootstrap re-sampling procedure was used, based on 100 bootstrap samples (sampling with replacement) of the same size as the original data set. The unadjusted and final adjusted models of the main analysis approaches were fit in each bootstrap sample. The stability of the relationship between the resulting treatment effect estimates (log HR from the unadjusted and adjusted models) were graphically displayed and described by linear regression.

### Statement of ethics

The EMPA-REG OUTCOME trial was designed and overseen by a steering committee that included academic investigators and employees of Boehringer Ingelheim. The trial was conducted in accordance with the principles of the Declaration of Helsinki and the International Conference on Harmonization Good Clinical Practice guidelines and was approved by local authorities. An independent ethics committee or institutional review board approved the clinical protocol at each participating center. A full list of participating sites and ethics committees can be found at https://www.nejm.org/doi/suppl/10.1056/NEJMoa1504720/suppl_file/nejmoa1504720_appendix.pdf. All the patients provided written informed consent before study entry.

## RESULTS

### Incidence of the composite kidney outcome

Of 7020 EMPA-REG OUTCOME participants, 6968 were eligible for inclusion in this analysis; 100/4645 (2.2%) participants in the pooled empagliflozin group and 86/2323 (3.7%) in the placebo group experienced an event of the composite kidney outcome.

### Conditions for potential mediators

The first mediation condition, the effect of empagliflozin on the potential mediators, was assessed for all investigated variables as previously reported [17]. To address the second condition, Table 1 presents the association of a 1-unit increase for each potential mediator variable with the risk of the composite kidney outcome. An increase in SBP, logUACR or uric acid, and a decrease in HDL, FFA, hematocrit, hemoglobin or albumin were significantly associated with an increased risk of the composite kidney outcome when analyzed as time-dependent updated mean or landmark Week 12 current change. For other factors, this applied only when using current change but not updated mean analyses, e.g. an increase in HbA1c, FPG, DBP or triglycerides was significantly associated with an increased risk of the composite kidney outcome only in the landmark Week 12 analysis using current change.

**Table 1:** Association of potential mediators with the risk of composite kidney outcome per 1-unit increase.

		HR (95% CI)^a^ per 1-unit increase of potential mediator	
		Time-dependent	Landmark Week 12 current
Physiological category	Potential mediator	updated mean	change from baseline
Glycemia	**HbA1c**, %	1.220 (0.992, 1.501)	**1.415 (1.132, 1.768)**
	**FPG**, mg/dL	0.999 (0.994, 1.005)	1.004 (1.000, 1.009)*
Vascular tone	**SBP**, mmHg	**1.018 (1.004, 1.033)**	**1.023 (1.012, 1.035)**
	**DBP**, mmHg	0.995 (0.969, 1.021)	**1.023 (1.001, 1.045)**
	Heart rate, BPM	1.021 (0.996, 1.046)	1.035 (0.994, 1.078)
Lipids	LDL, mg/dL	1.001 (0.995, 1.008)	1.003 (0.996, 1.010)
	**HDL**, mg/dL	**0.961 (0.935, 0.987)**	**0.972 (0.945, 0.999)**
	**Triglycerides**, mg/dL	1.001 (1.000, 1.003)	1.002 (1.000, 1.003)**
	**FFA**, mg/dL	**0.909 (0.873, 0.946)**	**0.917 (0.885, 0.950)**
Kidney	**logUACR**, mg/g	**2.085 (1.722, 2.524)**	**1.693 (1.397, 2.052)**
Adiposity	Weight, kg	0.989 (0.938, 1.042)	0.979 (0.911, 1.052)
	BMI, kg/m^2^	0.991 (0.857, 1.146)	0.958 (0.788, 1.164)
	Waist, cm	1.024 (0.989, 1.061)	1.009 (0.966, 1.055)
Volume status and hematopoiesis/oxygenation	**Hematocrit**, %	**0.741 (0.701, 0.783)**	**0.881 (0.824, 0.942)**
	**Hemoglobin**, g/dL	**0.424 (0.355, 0.507)**	**0.767 (0.623, 0.945)**
	**Albumin**, g/dL	**0.075 (0.033, 0.171)**	**0.233 (0.109, 0.498)**
Other	**Uric acid**, mg/dL	**1.898 (1.623, 2.219)**	**1.159 (1.001, 1.343)**

^a^Based on a Cox regression analysis of pooled treatment groups including the baseline value of the potential mediator as a covariate and a factor for treatment. *P = .0314. **P = .0181.

Variables in bold were considered to be associated with the composite kidney outcome as the 95% CI of the HR did not include 1, corresponding to a *P*-value <.05.

BPM, beats per minute.

### Cox regression analysis with time-dependent updated mean

**Figure 1:**
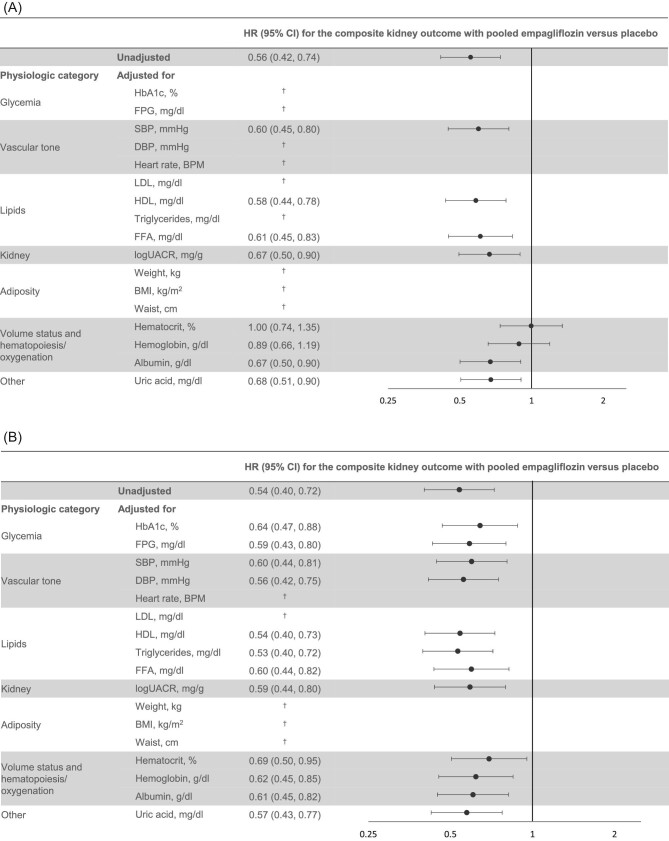
Univariable mediation analysis of risk of composite kidney outcome with empagliflozin versus placebo, (**A**) Cox regression with time-dependent updated mean and (**B**) Cox regression with landmark Week 12 current change from baseline. ^†^HR (95% CI) not presented for potential mediators that did not have a significant association with the composite kidney outcome. Cox proportional hazards regression analysis in participants treated with one or more doses of study drug. BPM, beats per minute.

**Figure 2:**
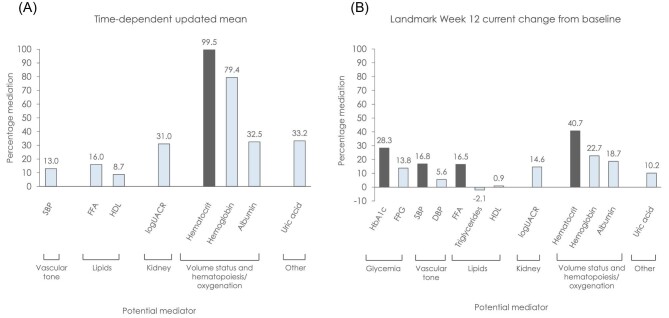
Percentage mediation from univariable analysis of the empagliflozin treatment effect on the composite kidney outcome, (**A**) Cox regression with time-dependent updated mean and (**B**) Cox regression with landmark Week 12 current change from baseline. Dark grey bars represent potential mediators maintained in multivariable model following sequential step-up procedure. Only potential mediators that were shown to have an effect on the composite kidney outcome are presented here, as only these can be considered mediators as per the traditional mediation analyses conditions.

**Figure 3:**
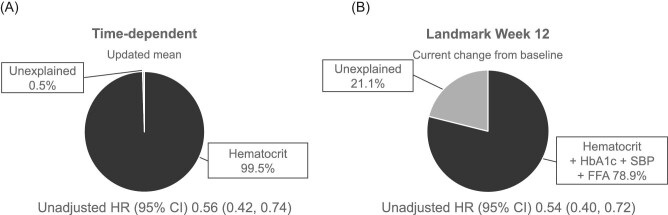
Percentage mediation of the empagliflozin treatment effect on the composite kidney outcome, (**A**) univariable Cox regression with time-dependent updated mean (multivariate analysis not performed due to overwhelming effect of hematocrit) and (**B**) multivariable Cox regression with landmark Week 12 current change from baseline. Unadjusted HR (95% CI) for empagliflozin versus placebo. Variables with the greatest mediating effect in the univariable analyses (one per physiologic category) were sequentially added using a step-up procedure in these univariable/multivariable analyses.

Based on a Cox regression analysis, the HR (95% CI) for the unadjusted effect of pooled empagliflozin versus placebo on the composite kidney outcome was 0.56 (0.42, 0.74), *P *< .001 (Fig. 1A). In univariable analyses adjusting for the updated mean and baseline value of the potential mediator, hematocrit appeared to be the strongest mediator of the empagliflozin treatment effect. With an estimated HR (95% CI) for the composite kidney outcome with pooled empagliflozin versus placebo of 1.00 (0.74, 1.35; Fig. 1A), hematocrit mediated 99.5% of the treatment effect (Figs 2A and 3A).

Due to the very strong mediation effect observed with hematocrit in the univariable analysis, no multivariable model was applied. Hemoglobin was the second strongest mediator, with 79.4% mediation of the empagliflozin treatment effect on the composite kidney outcome (Fig. 2A). Changes in uric acid, albumin, UACR, FFA or SBP mediated 33.2%, 32.5%, 31.0%, 16.0% and 13.0% of the treatment effect, respectively.

As hematocrit and hemoglobin had similarly strong impact on the treatment effect, we performed multivariable analyses using hemoglobin instead of hematocrit from the “volume status and hematopoiesis/oxygenation” mechanistic category. The combination of either uric acid or UACR with hemoglobin mediated 100% (Supplementary data, Fig. S1A), and the combination of FFA or SBP with hemoglobin mediated 90.2% and 90.6% of the treatment benefit, respectively.

### Cox regression analysis with landmark Week 12 current change from baseline

In the landmark Week 12 analyses, the estimated HR (95% CI) for the unadjusted treatment effect on the composite kidney outcome was 0.54 (0.40, 0.72), *P *< .001 (Fig. 1B). In univariable analyses using current change from baseline at Week 12, again hematocrit acted as the strongest mediator, but with an estimated HR (95% CI) for the composite kidney outcome with empagliflozin versus placebo of 0.69 (0.50, 0.95; Fig. 1B), resulting in 40.7% mediation (Fig. 2B). Changes in HbA1c, hemoglobin, albumin, SBP and FFA mediated 28.3%, 22.7%, 18.7%, 16.8% and 16.5%, respectively. Other factors with a minor mediation effect included UACR, FPG and uric acid, mediating 14.6%, 13.8% and 10.2% of the empagliflozin treatment effect, respectively. The other factors investigated, including DBP, HDL cholesterol, and triglycerides had no or negligible effects in these analyses.

In a multivariable model using current change at Week 12, the combination of hematocrit, HbA1c, SBP and FFA mediated 78.9% of the treatment effect (Fig. 3B).

### Sensitivity analysis: Cox regression analysis with time-dependent current change

When using the current change from baseline of the respective potential mediator as a time-dependent covariate, results were consistent with the previous results when using updated mean as the time-dependent covariate (Supplementary data, Fig. S2A). Hematocrit acted as the strongest mediator, with an estimated HR (95% CI) for composite kidney outcome with empagliflozin versus placebo of 0.93 (0.70, 1.25), 88.0% mediation; hemoglobin was the second strongest, accounting for 70.4% mediation. Factors with some mediation effect included uric acid, albumin, UACR and HDL cholesterol, mediating 38.9%, 24.7%, 12.7% and 11.0%, respectively (Supplementary data, Fig. S2A). Other factors including SBP and FFA had no or negligible mediation effects.

As hematocrit alone did not fully mediate the treatment effect in the univariable analysis using current change, a multivariable analysis was performed. The combination of hematocrit with uric acid mediated 100.0% of the treatment effect (Supplementary data, Fig. S3A).

As hematocrit and hemoglobin had similarly strong impact on the treatment effect when using current change, we performed additional multivariable analyses using hemoglobin instead of hematocrit. In the multivariable model, 81.0% of the treatment benefit was mediated by hemoglobin in combination with UACR, 77.9% with HDL and 95.0% with uric acid (Supplementary data, Fig. S1B).

### Sensitivity analysis: Cox regression analysis with landmark Week 12 updated mean

When using the updated mean at Week 12, results were generally consistent with the analysis using current change at Week 12 in landmark analyses. Hematocrit acted as the strongest mediator, with an estimated HR (95% CI) for composite kidney outcome with empagliflozin versus placebo of 0.68 (0.50, 0.94), accounting for 38.5% mediation (Supplementary data, Fig. S2B). Changes in HbA1c, hemoglobin, FFA, albumin and SBP mediated 26.3%, 21.1%, 17.1%, 16.0% and 15.2%, respectively. Other factors with a minor mediation effect included uric acid and UACR, mediating 11.0% and 7.9% of the empagliflozin treatment effect, respectively.

In line with the results using the current change, in the multivariable Week 12 landmark model using the updated mean, the combined effect of hematocrit, HbA1c, SBP and FFA mediated most of the treatment effect (77.1%; Supplementary data, Fig. S3B).

### Sensitivity analysis: bootstrap resampling

Results of stability analyses based on bootstrap resampling are shown in Supplementary data, Fig. S4 for time-dependent analysis approach including the updated mean of the mediators and Supplementary data, Fig. S5 for the landmark Week 12 analysis approach including the current change from baseline of the mediators. In both approaches, the results on mediation of the treatment effect were stable over the bootstrap samples, as there were high correlations between the unadjusted and the adjusted logHR over the bootstrap samples, the estimated slopes of the regression lines were near 1, and the estimated intercepts of the regression lines were of similar magnitude as the differences between the unadjusted and adjusted logHR from the original data. The medians of the proportion mediated estimated in the 100 bootstrap samples were also similar to the proportion mediated estimated from the models in the original data.

## DISCUSSION

In this *post hoc* mediation analysis of the EMPA-REG OUTCOME trial, we found that regardless of the statistical model applied, changes in hematocrit and hemoglobin levels were the strongest mediators of the empagliflozin treatment benefit on the composite kidney outcome of first sustained eGFR reduction of ≥40% from baseline, initiation of renal replacement therapy, or death due to kidney disease. For example, in analysis that reflects the cumulative effect of all prior values of the variable on the risk of outcome (Cox regression analysis with time-dependent updated mean), hematocrit alone seemed to mediate almost all the observed treatment effect on the composite kidney outcome. The magnitude to which variables contributed to the empagliflozin treatment effect on the composite kidney outcome varied among the different statistical models applied. When using landmark analyses (at Week 12) of the impact of initial change of the variable, the mediation effect of hematocrit or hemoglobin were lower and more comparable to other factors (such as HbA1c, albumin, UACR, SBP, FFA and uric acid) compared with estimations using the time-dependent covariates over the observation period.

Overall, our findings were consistent with previously reported mediation analyses from EMPA-REG OUTCOME of cardiovascular mortality [17] and adverse heart failure outcomes [18], in which changes in hematocrit and hemoglobin were shown to mediate the majority of the treatment benefit of empagliflozin. Furthermore, the results of this analysis are supportive of the findings of a mediation analysis of the CANagliflozin cardioVascular Assessment Study (CANVAS) [15], in which Li *et al.* reported that markers of volume status and oxygenation/erythropoiesis (hematocrit and hemoglobin) were strong mediators of the canagliflozin treatment effect on a composite kidney outcome (sustained 40% decrease in eGFR, end-stage kidney disease or death due to kidney disease) in all statistical models applied. Post-randomization changes in urate, UACR, albumin and SBP mediated the canagliflozin treatment effect to a lesser extent than hematocrit and hemoglobin, and serum bicarbonate, lipid parameters and HbA1c exhibited negligible effects. The multivariable model showed that the combination of erythrocytes and serum urate mediated 103.0% of the treatment benefit of canagliflozin. Additionally, in their sensitivity analysis assessing the impact of the early changes of potential mediators instead of the average post-randomization level, the magnitude of the mediating effect was somewhat smaller across all parameters. This appears to be in line with the findings of the present study, mirroring the higher contribution of volume/oxygenation markers found in the time-dependent analyses, compared with the more evenly distributed contribution across various pathobiological mechanistic factors in the landmark Week 12 analysis.

Thus, the long-term impact of hematocrit and hemoglobin on the composite kidney outcomes appears to be stronger than the impact of the initial change up to Week 12. The initial changes may mainly reflect hemoconcentration from plasma volume reduction due to urinary sodium and water losses, whilst long-term changes likely represent mainly erythropoiesis via enhanced erythropoietin secretion by the kidney. Clinical studies have shown an increase in serum erythropoietin levels as early as 4 weeks following initiation of treatment with SGLT2 inhibitors [20, 21]. As a direct consequence of enhanced hematopoiesis, hemoglobin and hematocrit can be regarded as markers of oxygenation of organs. Several mechanisms have been suggested that might, at least in part, explain the predominant role of those oxygenation markers in the empagliflozin kidney benefits observed in the EMPA-REG OUTCOME trial. One possible mechanism is simply improved oxygenation, with subsequent alleviation of renal hypoxia. Hypoxia injury is felt to be a final common pathway to end-stage kidney disease [22], and studies have demonstrated the presence of intrarenal hypoxia in patients with diabetes [23]. Furthermore, a blood oxygenation level–dependent magnetic resonance imaging study published in 2018 of patients with chronic kidney disease, including those with diabetic kidney disease, showed that kidney tissue hypoxia is predictive of adverse kidney outcomes [24].

The causes of hypoxia in diabetic kidney disease are multifactorial including changes of peritubular capillary flow due to the imbalance of vasoactive substances, and an increase of oxygen demand due to oxidative stress [25]. SGLT2 inhibitors have been shown to increase peritubular capillary flow via post-glomerular vasodilation [26], improve mitochondrial function and reduce oxidative stress in the kidneys [27]. They have also been shown to enhance erythropoiesis via activation of the hypoxia-inducible factor (HIF) pathway in a manner that favorably affects the course of diabetic kidney disease [28–32]. A recent study of a single-cell atlas showed induction of hypoxia responses and fasting mimicry as the early effects of SGLT2 inhibitors on the S1 segment of the proximal tubule [33]. Notably, the HIF pathway (involving the HIF-1α and HIF-2α transcription factors) is a master regulator of adaptive responses to hypoxia such as erythropoiesis [34, 35]. Some of the kidney effects of SGLT2 inhibitors may be through changes in the HIF system [32]. Studies have demonstrated that chronic hypoxia plays a crucial role in progression of kidney disease [36–38]. It has been proposed that by blocking proximal tubular sodium reabsorption, SGLT2 inhibitors reduce oxygen demand and alleviate hypoxia, which alleviates the tubulointerstitial injury [39]. Additionally, SGLT2 inhibitors enhance nutrient deprivation signaling as a result of the loss of calories in the urine, leading to upregulation of adenosine monophosphate-activated protein kinase (AMPK) and sirtuin-1 (SIRT1) [40, 41], which additionally suppress HIF-1α [42–44].

Some limitations of the present study should be considered. As this was a *post hoc* study, the results can only be regarded as hypothesis generating. Also, the finding of a statistical mediation effect does not prove that improvement of the outcome directly resulted from empagliflozin-induced changes in that covariate. The covariate could instead be linked to another true but unmeasured mediator, for example. Moreover, it also cannot be inferred that similar changes in these variables, achieved with approaches other than empagliflozin treatment, will have similar effects on kidney outcomes. The sensitivity of a mediation analysis is furthermore dependent on which variables were measured in the study. Thus, it is possible that other effects of empagliflozin might have had an effect on kidney outcomes but were not assessed in this analysis as they were not measured during the EMPA-REG OUTCOME trial.

In conclusion, our finding that the most important mediators of the kidney benefits observed with empagliflozin in the EMPA-REG OUTCOME trial were hematocrit and hemoglobin is in line with mediation analyses on cardiovascular mortality and heart failure hospitalization outcomes with empagliflozin. The predominance of these markers of erythropoiesis and oxygenation as mediator of the empagliflozin kidney benefits, particularly when assessed in a time-dependent manner, may suggest an erythropoietic mechanism of action, leading to alleviation of hypoxia in the kidneys. Other factors that register as moderate mediators include uric acid, SBP and FFA. However, further studies are required to validate the underlying mechanisms suggested by the findings of this study.

## Supplementary Material

gfae032_Supplemental_File

## Data Availability

To ensure independent interpretation of clinical study results and enable authors to fulfil their role and obligations under the ICMJE criteria, Boehringer Ingelheim grants all external authors access to relevant clinical study data. In adherence with the Boehringer Ingelheim Policy on Transparency and Publication of Clinical Study Data, scientific and medical researchers can request access to clinical study data, typically 1 year after the approval has been granted by major Regulatory Authorities or after termination of the development program. Researchers should use the https://vivli.org/link to request access to study data and visit https://www.mystudywindow.com/msw/datasharing for further information.
