# Supplementary material for: How do SGLT2 inhibitors protect the kidney? A mediation analysis of the EMPA-REG OUTCOME trial
Source: Nephrol Dial Transplant. 2024 Feb 6;39(9):1504–13. doi: 10.1093/ndt/gfae032 (PMC11361804; doi:10.1093/ndt/gfae032)
Supplement: gfae032_Supplemental_File [file gfae032_supplemental_file.docx]

Supplementary Material

[**Supplementary Figure 1** Percentage mediation of the empagliflozin treatment effect on the composite kidney outcome. 2](#_Toc124950447)

[**Supplementary Figure 2** Univariable mediation analysis and percentage mediation of risk of composite kidney outcome with empagliflozin versus placebo 3](#_Toc124950448)

[**Supplementary Figure 3** Sensitivity analysis: Percentage mediation of the empagliflozin treatment effect on the composite kidney outcome 5](#_Toc124950449)

[**Supplementary Figure 4** Statistical stability of mediation of treatment effect in univariable model for time-dependent updated mean. 6](#_Toc124950450)

[**Supplementary Figure 5** Statistical stability of mediation of treatment effect in multivariable model for landmark Week 12 current change from baseline. 7](#_Toc124950451)

**Supplementary Figure 1** Percentage mediation of the empagliflozin treatment effect on the composite kidney outcome. Multivariable Cox regression with time-dependent covariates including hemoglobin (in lieu of hematocrit) using the (A) updated mean and B) using current change from baseline


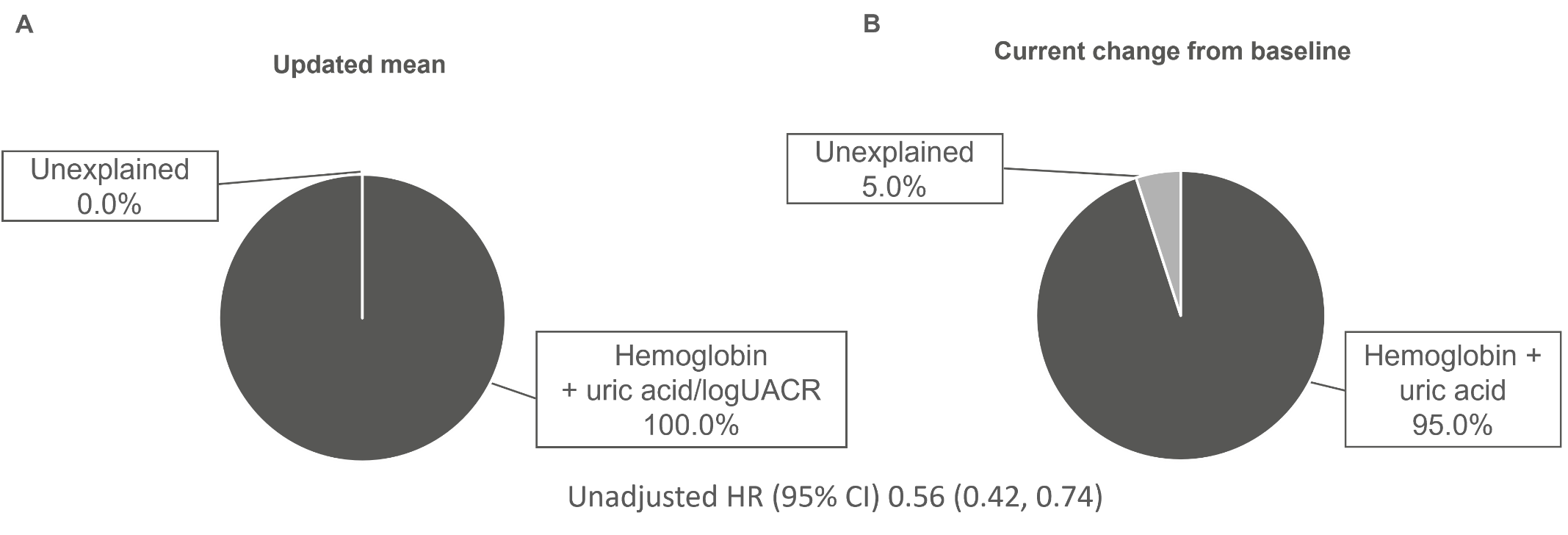


Multivariable models when hemoglobin instead of hematocrit is used as representative from mechanistic category volume status and hematopoiesis/oxygenation, while for all other mechanistic categories the variables with the greatest mediating effect in the univariable analyses (one per physiologic category) were sequentially added using a step-up procedure in these multivariable analyses. Unadjusted HR (95% CI) for empagliflozin versus placebo.

CI, confidence intervals; HR, hazard ratio

**Supplementary Figure 2** Univariable mediation analysis and percentage mediation of risk of composite kidney outcome with empagliflozin versus placebo, A) Cox regression with time-dependent current change from baseline, B) Cox regression with landmark Week 12 updated mean


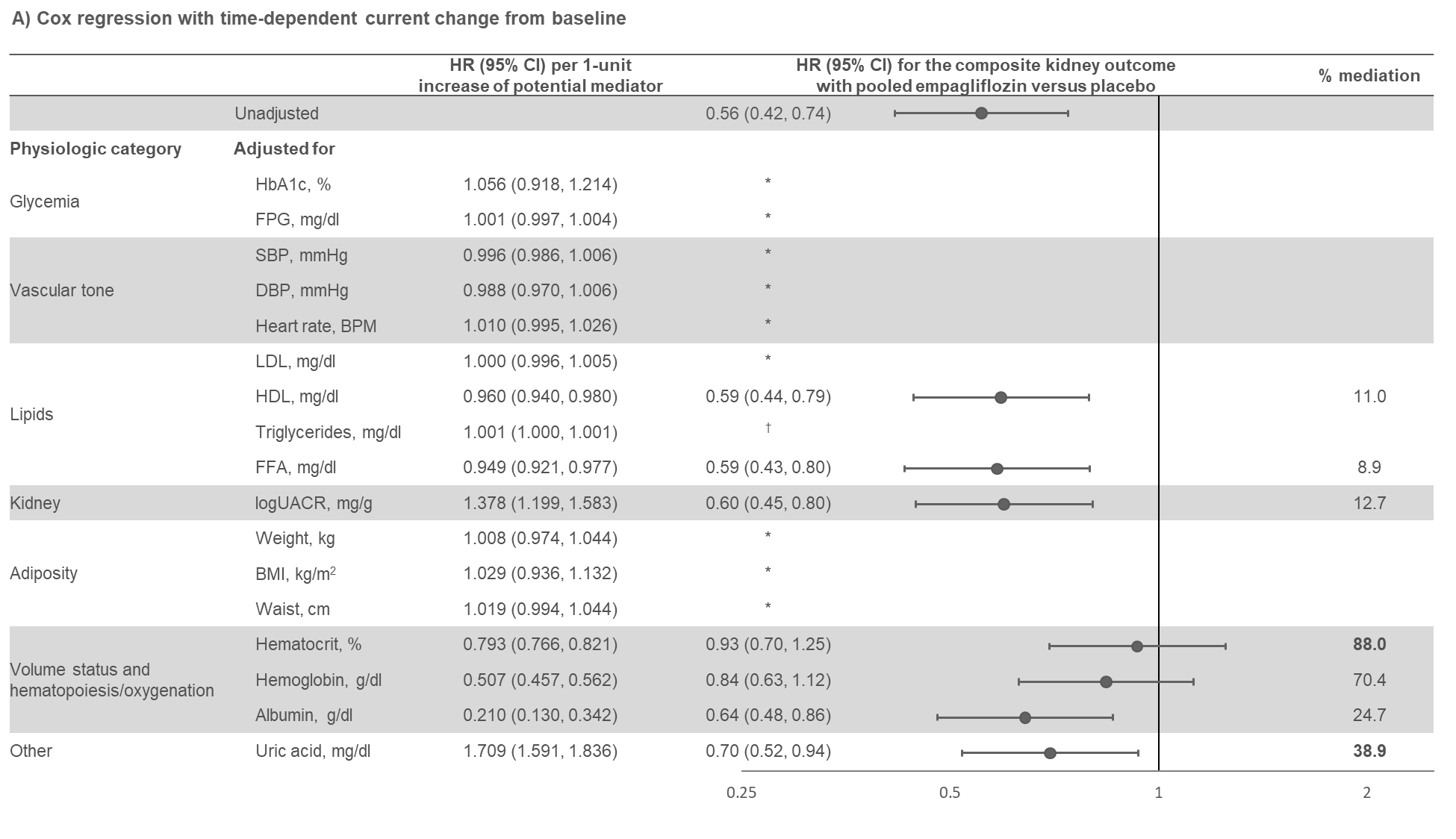


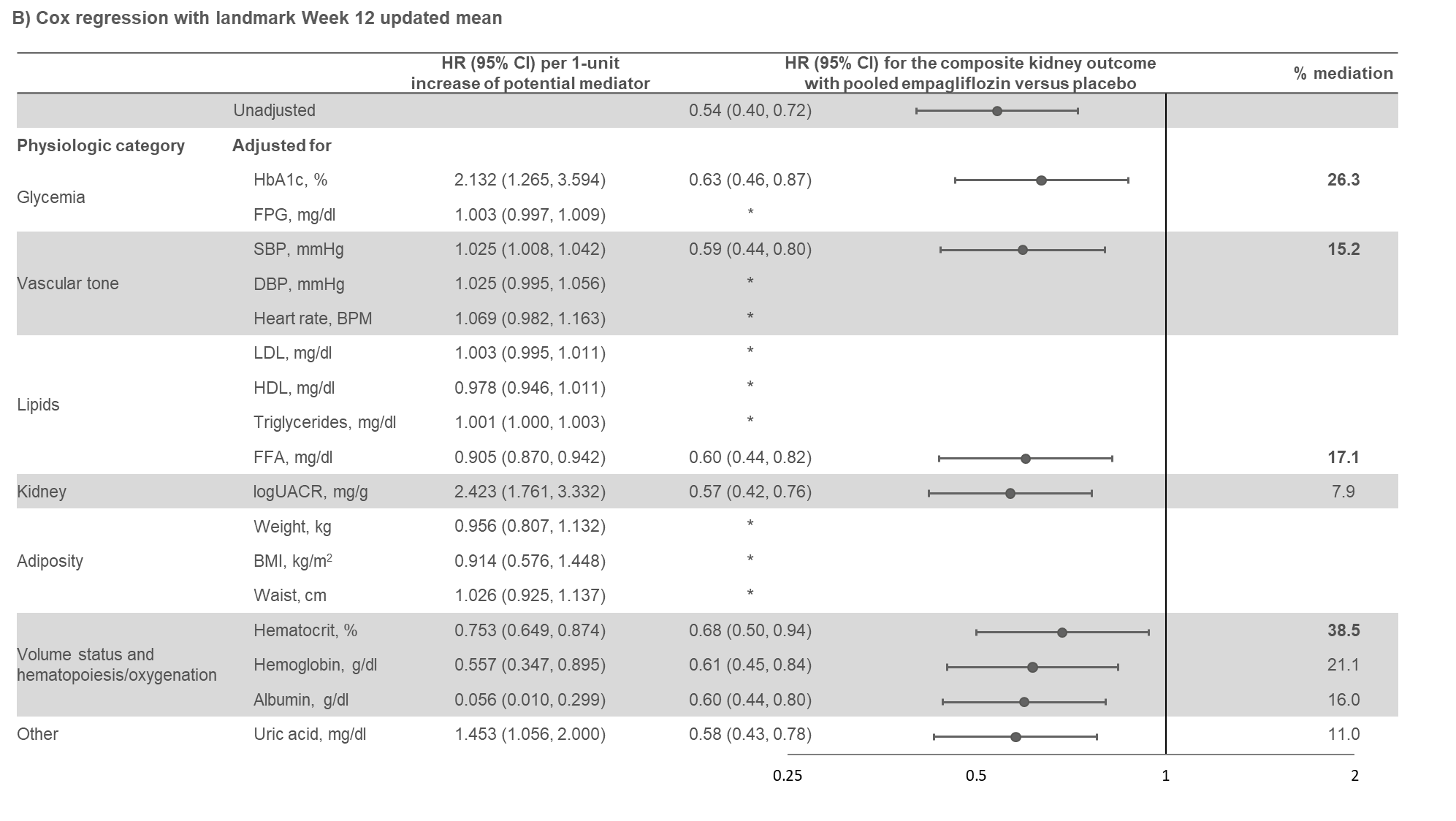


*HR (95% CI) not presented for potential mediators that did not have a significant association with the composite kidney outcome. Percent mediation for the factors that were subsequently included in the multivariable analysis are given in bold font.
BMI, body mass index; BPM, beats per minute; CI, confidence intervals; DBP, diastolic blood pressure; FFA, free fatty acids; FPG, fasting plasma glucose; HbA1c, glycated hemoglobin; HDL, high density lipoprotein cholesterol; HR, hazard ratio; LDL, low density lipoprotein cholesterol; SBP, systolic blood pressure; UACR, urine albumin-to-creatinine ratio.

**Supplementary Figure 3** Sensitivity analysis: Percentage mediation of the empagliflozin treatment effect on the composite kidney outcome, multivariable A) Cox regression with time-dependent current change from baseline, B) Cox regression with landmark Week 12 updated mean


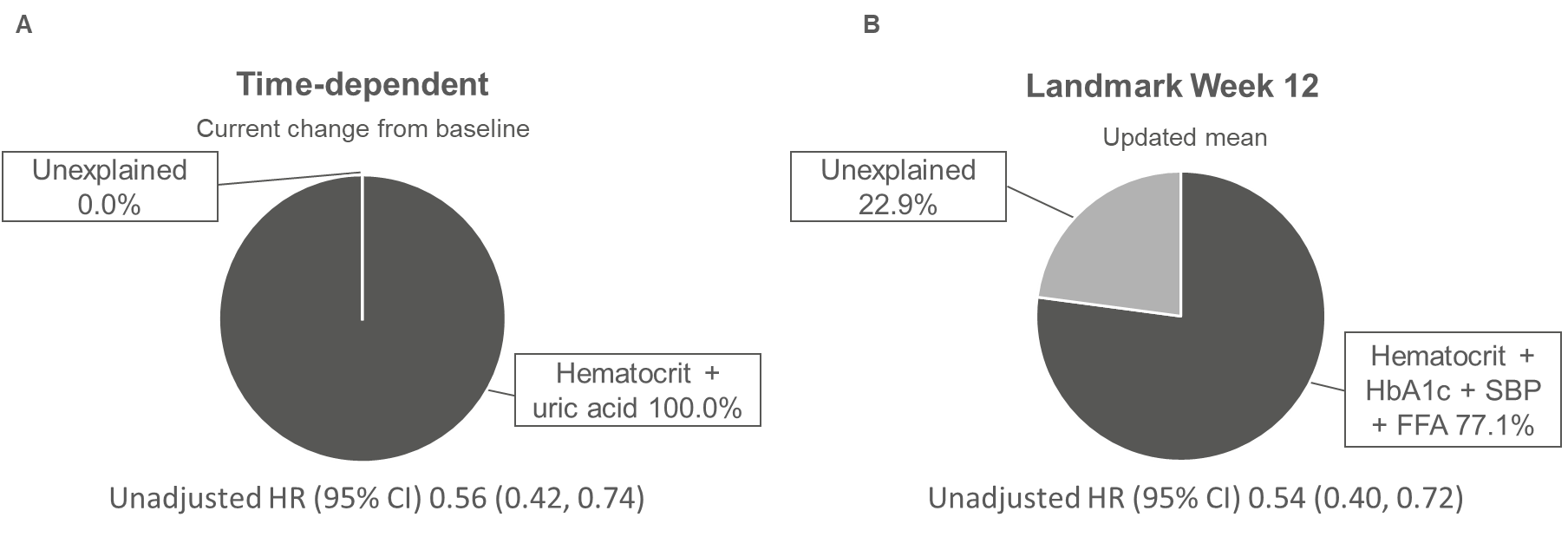


Unadjusted HR (95% CI) for empagliflozin versus placebo. Variables with the greatest mediating effect in the univariable analyses (one per physiologic category) were sequentially added using a step-up procedure in these multivariable analyses.
CI, confidence intervals; FFA, free fatty acids; HbA1c, glycated hemoglobin; HR, hazard ratio; SBP, systolic blood pressure.

**Supplementary Figure 4** Statistical stability of mediation of treatment effect in univariable model for time-dependent updated mean. The unadjusted and adjusted* models were fit in each of 100 bootstrap samples. In the original data set, the logHR of treatment with empagliflozin vs placebo in the adjusted analysis was -0.0030 (difference vs the logHR of the unadjusted analysis of -0.5896 was 0.5866) and the resulting proportion mediated was 99.5%. The results on mediation of the treatment effect were stable over the bootstrap samples, represented by the intercept of 0.50 estimated from linear regression, corresponding to the estimated difference between unadjusted and adjusted logHR. The median of the proportion mediated estimated in the 100 bootstrap samples was 97.9%.


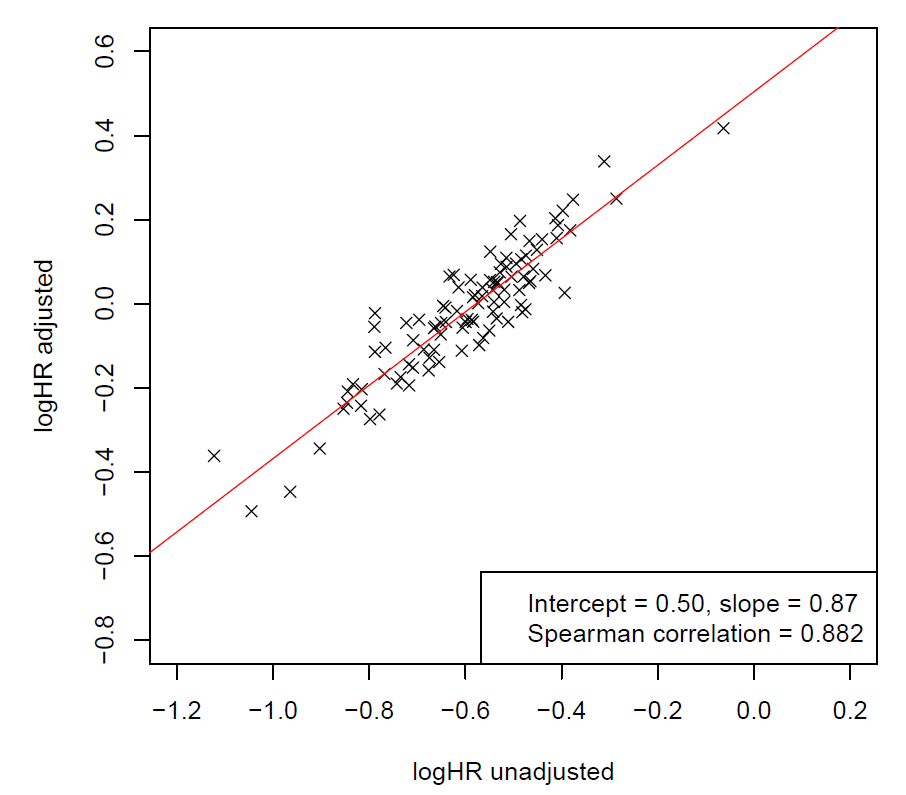


*Adjusted model includes factors for pooled treatment groups, baseline hematocrit and time-dependent updated mean of hematocrit

**Supplementary Figure 5** Statistical stability of mediation of treatment effect in multivariable model for landmark Week 12 current change from baseline. The unadjusted and adjusted* models were fit in each of 100 bootstrap samples. In the original data set, the logHR of treatment with empagliflozin vs placebo in the adjusted analysis was -0.1295 (difference vs the logHR of the unadjusted analysis of -0.6184 was 0.4888) and the resulting proportion mediated was 78.9%. The results on mediation of the treatment effect were stable over the bootstrap samples, represented by the intercept of 0.48 estimated from linear regression, corresponding to the estimated difference between unadjusted and adjusted logHR. The median of the proportion mediated estimated in the 100 bootstrap samples was 80.9%.


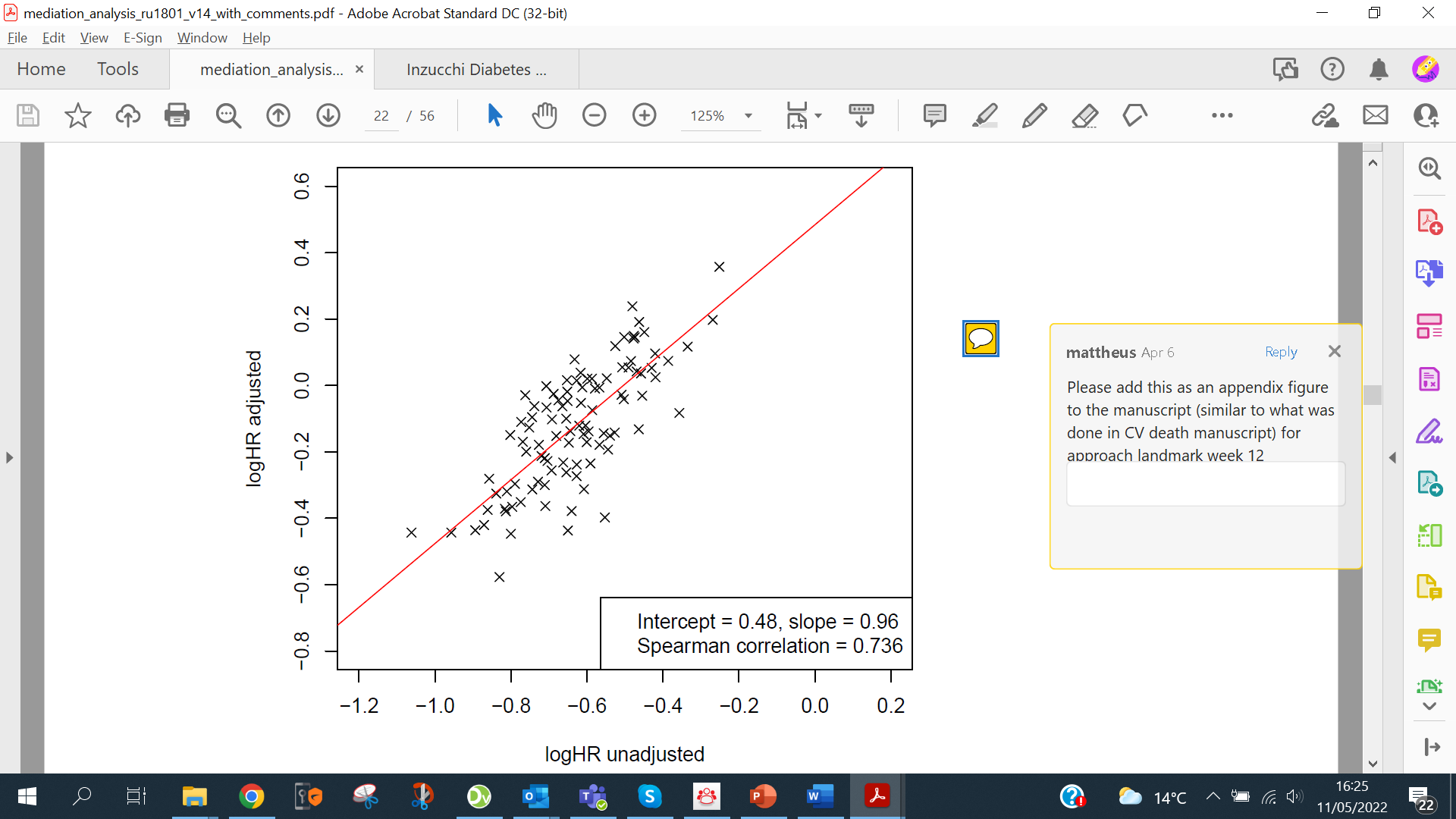


*Adjusted model includes factors for pooled treatment groups, baseline value and current change from baseline at week 12 in hematocrit, HbA1c, SBP, FFA
